# Analysis of histopathology and changes of major cytokines in the lesions caused by *Mycoplasma ovipneumoniae* infection

**DOI:** 10.1186/s12917-023-03829-4

**Published:** 2023-12-15

**Authors:** Jidong Li, Can Chen, Le Gao, Lingling Wang, Wei Wang, Jinhua Zhang, Zhenxing Gong, Jiandong Wang, Yanan Guo

**Affiliations:** 1 School of Agriculture, Ningxia University, Yinchuan, 750021 Ningxia China; 2 Institute of Animal Science, Ningxia Academy of Agricultural and Forestry Sciences, Yinchuan, 750002 Ningxia China

**Keywords:** Sheep, *Mycoplasma ovipneumoniae*, Histopathological analysis, Immunohistochemistry, Cytokine

## Abstract

**Background:**

*Mycoplasma ovipneumoniae* (*M. ovipneumoniae*) is one of the main pathogens of sheep pneumonia, causing a series of clinical symptoms, such as depression, anorexia, hyperthermia, cough, dyspnea, and tract secretions. In recent years, the prevalence of *M. ovipneumoniae* pneumonia has become increasingly serious in sheep farms in Ningxia, China, leading to the death of sheep, and causing significant economic losses. In this study, the pathological organs infected by *M. ovipneumoniae* were collected to observe histopathological change, to determine the tissue localization of *M. ovipneumoniae*, and to analyze the cytokine changes, which lays a basis for the diagnosis and pathogenesis of *M. ovipneumoniae* disease.

**Results:**

In this study, *M. ovipneumoniae* was detected in 97 of 105 samples collected from 13 large-scale sheep farms for nucleic acid by PCR. One representative isolate per farm was isolated from 13 farms. The lesions caused by *M. ovipneumoniae* were mainly in the trachea, bronchus, and lung, including necrosis of tracheal mucosal epithelial cells, disintegration of some epithelial cells, edema of mucosal lamina propria, with inflammatory cell infiltration, cytoplasmic vacuolization of epithelial cells of bronchial mucosa, massive infiltration of inflammatory cells in the alveolar space of lung, necrosis and hyperplasia of alveolar epithelial cells. Immunohistochemical analysis showed that the proportion of *M. ovipneumoniae* positive area in the lung was the largest, followed by that in the bronchus and trachea. Compared to healthy animals, diseased animals exhibited up-regulated gene expression levels of IL-1β, IL-6, and NF-κB in the trachea, bronchus, and lungs. In contrast, the expression of IL-10, IL-12, and IFN-γ was primarily limited to the trachea and bronchus. The expression of IL-1β showed differential patterns across different lung regions, with variations observed among lung lobes. Additionally, other cytokines consistently showed significant up-regulation specifically in the bronchus.

**Conclusions:**

*M. ovipneumoniae* is primarily found in the lungs of infected individuals. NF-κB, an essential transcription factor, is involved in the regulation of IL-1β transcription. IL-12 may enhance the cytotoxic function of natural killer cells during *M. ovipneumoniae* infection. Those findings demonstrate the distinct expression profiles of cytokines in various anatomical sites throughout disease progression, suggesting the potential role of bronchial tissue as a major site of immune response.

**Supplementary Information:**

The online version contains supplementary material available at 10.1186/s12917-023-03829-4.

## Background

*Mycoplasma ovipneumoniae* (*M. ovipneumoniae*) can cause respiratory diseases in sheep and goats. The main clinical symptoms are cough, asthma, and dyspnea after exercise. Pulmonary interstitial hyperplasia, pleural adhesions, and serous or fibrinous exudate in the thoracic cavity are found in diseased sheep by means of pathological dissection [1]. *M. ovipneumoniae* can be isolated from the lungs, the trachea, and nasal passages of diseased sheep, and from the respiratory tract of healthy sheep [2]. The *M. ovipneumoniae* infection rate and fatality rate of sheep aged from 1–3 months are higher than that of sheep aged other months. The immune evasion of mycoplasma enables the pathogen to exist in the host for a long time, leading to subclinical symptoms in infected animals [2, 3]. The pathogen is transmitted in the environment primarily through respiratory tract secretions. If tolerance of infected sheep is established, the covert infected sheep will become a potential source of infection to healthy animals [1, 4].

The main methods to detect *M. ovipneumoniae* are isolation and identification of pathogen, and nucleic acid amplification of specific genes [5]. These methods have their advantages, but they are not able to detect antigen localization in the tissues. *M. ovipneumoniae* infection leads to non-specific changes in pathological dissection and histopathology, mainly in the respiratory system [6]. Immunoblotting performed on the isolates or tissue samples may provide information about antigens [7]. The corresponding antigen or antibody is tested qualitatively and quantitatively using visible chromogenic reagents. Immunohistochemistry is used to study the location and distribution of pathogens in organs, tissues, and cells, to provide the basis for the diagnosis of pathological lesions [8]. Studies show that *M. ovipneumoniae* mainly colonizes in the lungs, trachea, and bronchus, and is located at epithelial cells and cytoplasm of trachea, bronchus, and lung [5, 9]. These findings can be used in auxiliary diagnosis.

The *M. ovipneumoniae* antigens enter the respiratory tract and adhere to respiratory epithelial cells. They stimulate an innate immune response in the host. As a large number of pathogens invade the target cells to reproduce and grow, they stimulate the host to produce a great quantity of macrophages, neutrophils, lymphocytes, Natural killer (NK) cells, and other immunocytes. The immunocytes can release all kinds of cytokines, and these cytokines bind to their receptors to mediate and regulate the immune response and inflammatory response [10–12]. The cytokines have the characteristics of overlap, pleiotropy, network, cooperativity, and antagonism. They can interact with each other and influence the formation of various immunoglobulins, complement, and acute phase proteins. Together with these proteins, they form a complex immune network [11, 13]. However, there is rare research on the changes of main immune factors and the main survival sites in sheep infected with *M. ovipneumoniae*. Therefore, studying the histopathological and immune factor changes induced by *M. ovipneumoniae* lays a basis for the diagnosis and pathogenesis of *M. ovipneumoniae* disease.

In this study, a total of 105 samples were collected from 13 large-scale farms in Ningxia to isolate and identify *M. ovipneumoniae*. 5 diseased and dead sheep from 5 farms among 13 sheep farms were autopsied and the pathological organs were collected to observe histopathological change, determine the tissue localization of *M. ovipneumoniae*, analyze the cytokine changes in the target organs. This study lays a foundation for the diagnosis and pathological damage mechanism of *M. ovipneumoniae*.

## Results

### The results of pathological anatomy

**Fig. 1:**
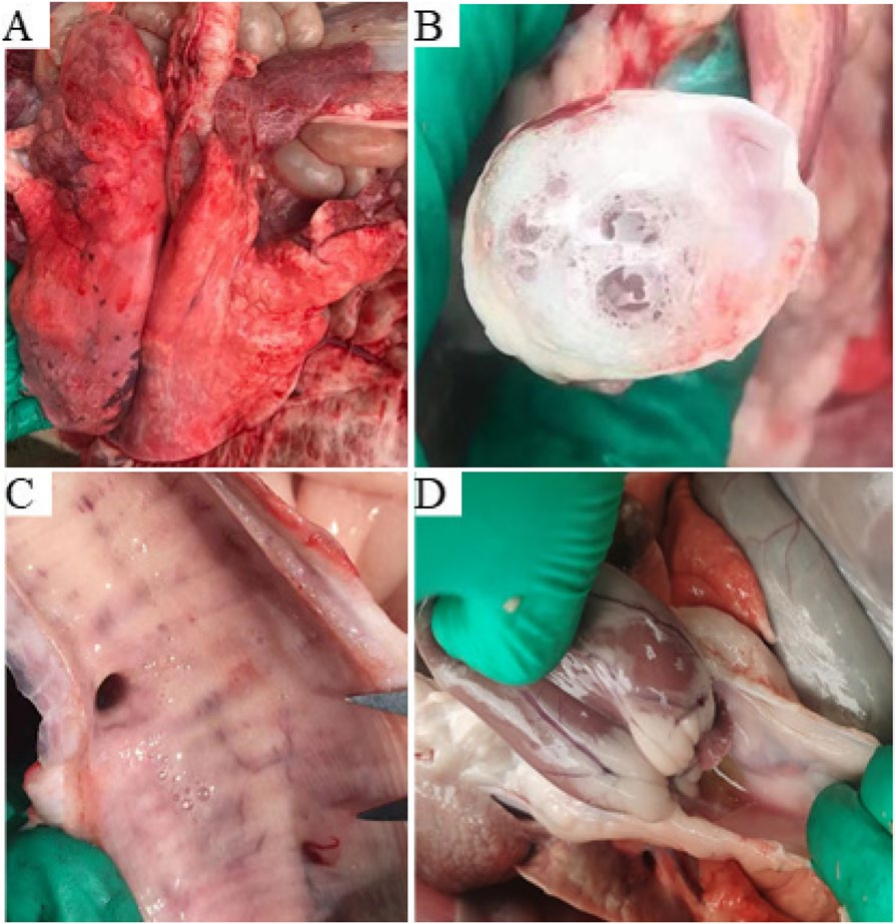
Pathological autopsy results. **A** The lungs adhered to the chest wall and there were substantial fleshy changes in both lungs. **B** There was a lot of white mucus in the bronchus. **C** There was a lot of white mucus in the trachea. **D **Yellowish fluid was present in the pericardium

After 5 diseased and dead sheep were dissected, lesions were mainly in the lungs and trachea, with serious substantive fleshy lesions in the bilateral cranial lobe of the lungs (Fig. 1A), and there is a lot of foam-like mucus in the bronchus (Fig. 1B), and a lot of white mucus in the trachea (Fig. 1C). Light yellow effusion was found in the pericardium (Fig. 1D). The autopsy changes of different viscera tissues in 5 sheep were consistent.

### The results of isolation, PCR identification, and molecular evolution analysis of *M. ovipneumoniae* isolates

**Fig. 2:**
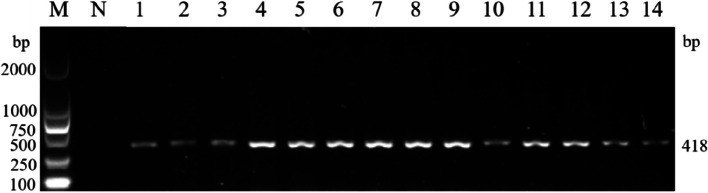
PCR result of *M. ovipneumoniae* isolates. M DL1 000 DNA marker. N Negative control. 1 Positive control. 2 ~ 14 Isolated strains fragment amplified

A total of 105 samples were collected from 13 large-scale sheep farms and 97 samples were identified as *M. ovipneumoniae* samples for nucleic acid by PCR. One representative isolate per farm was isolated from 13 farms. The genomic DNA of 13 isolates were amplified by PCR using *M. ovipneumoniae*-specific primers, and the expected product of 418 bp was obtained (Fig. 2).

**Fig. 3:**
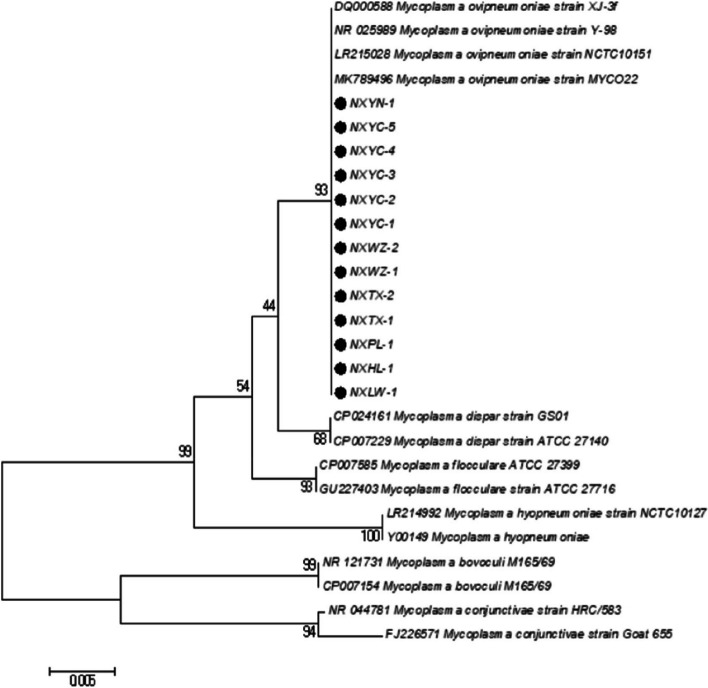
Phylogenetic tree based on 16S rRNA gene sequence of *M. ovipneumoniae* isolates

Phylogenetic tree based on 16S rRNA gene sequence showed that all 13 isolates belong to *M. ovipneumoniae* (Fig. 3). There was the highest homology between 13 M*. ovipneumoniae* isolates with *M. ovipneumoniae* MYC022 (MK789496), *M. ovipneumoniae* NCTC10151 (LR215028), *M. ovipneumoniae* XJ-3f, *M. ovipneumoniae* Y-98 (NR025989). The relation between 13 M*. ovipneumoniae* isolates with *M. bovoculi* M165/69 (NR121721), *M. bovoculi* M165/69 (CP007154), *M. conjunctivae* HRC/583 (NR044781), and *M. conjunctivae* Goat 655 (FJ226571) was the most distant relatively.

### Histopathological results

**Fig. 4:**
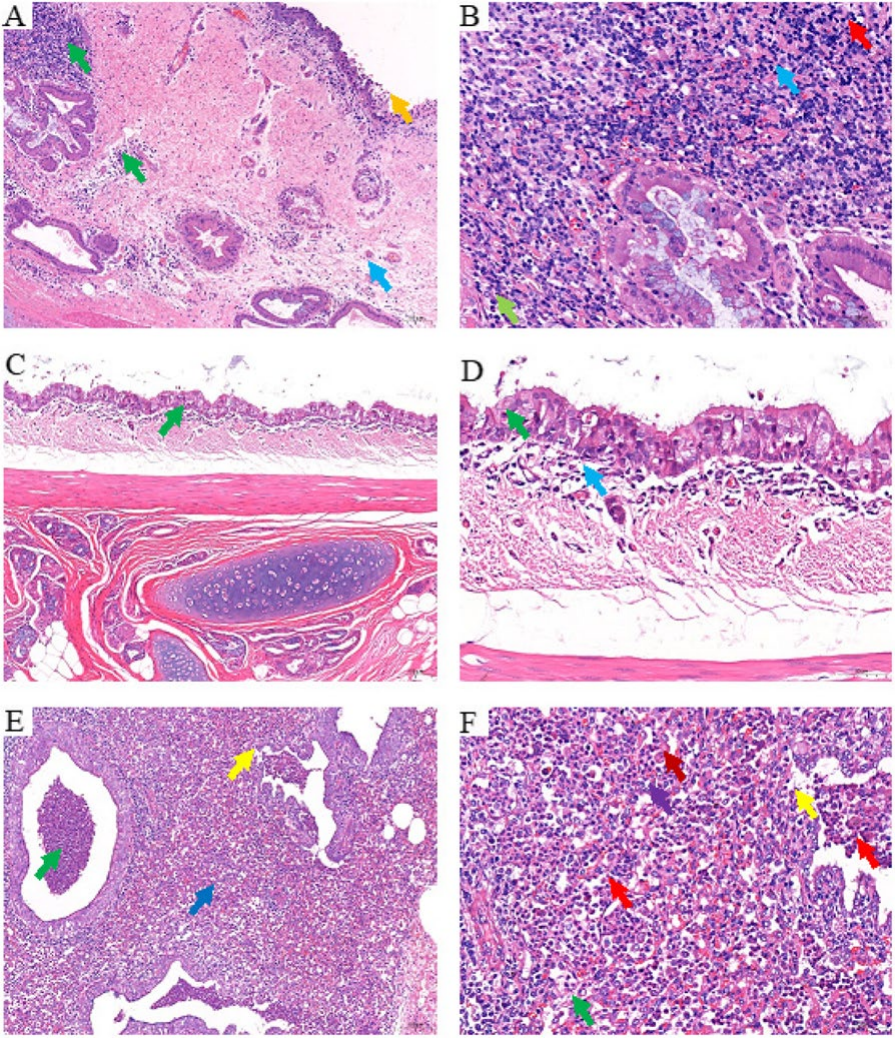
Histopathological analysis results of different tissues (Hematoxylin–eosin). **A** Histopathological analysis of trachea (100 ×), necrosis and shedding of mucosal epithelial cells (**↑**), lamina propria edema (**↑**), inflammatory cells infiltration (**↑**). **B** Histopathological analysis of trachea (400 ×), lymphocyte infiltration (**↑**), plasma cell infiltration (**↑**), neutrophil infiltration (**↑**). **C** Histopathological analysis of bronchus (100 ×), inflammatory cells infiltration (**↑**). **D** Histopathological analysis of bronchus (400 ×), necroptosis of epithelial cells (**↑**), lymphocyte infiltration (**↑**). **E** Histopathological analysis of lung (100 ×), inflammatory cell infiltration in the bronchial lumen (**↑**), shedding of bronchial epithelial cells (**↑**), increased in the alveolar space (**↑**). **F** Histopathological analysis of lung (400 ×), necrotic shedding of bronchial epithelial cells (**↑**), alveolar epithelial cell necrosis (**↑**), hyperplasia (**↑**), neutrophil infiltration (**↑**), increased macrophages (**↑**)

The collected visceral tissues were paraffin sectioning and H·E staining for microscopic examination. The histopathological changes of different visceral tissues in 5 sheep were consistent. The results were as follows:

Trachea: mucosal epithelial cells were necrotic and shed, some epithelial cells disintegrated, and the structure was blurred. Mucosal lamina propria became edema with a small amount of inflammatory cell infiltration. The intercellular space was widened, and there was an uneven amount of inflammatory cell infiltration in the stroma, some of which formed inflammatory infiltration foci. The inflammatory cells were mainly lymphocytes and plasma cells, and a small number of neutrophils were also observed. (Fig. 4A, B).

Bronchus: a few cells of the mucosa epithelium were necrotic, and the cytoplasm of the epithelium cells was vacuolated and the nucleus was contracted. A small number of inflammatory cells, mainly lymphocytes, can be seen locally in the superficial layer of lamina propria (Fig. 4C, D).

Lung: local alveolar septum was thickened, part of the bronchial epithelial cells was necrotic and exfoliated, and exfoliated cell fragments and inflammatory cell infiltration were found in the bronchial lumen, mainly segmented neutrophils. A large number of inflammatory cells were infiltrated in the alveolar space, mainly neutrophils, with an increased number of macrophages, and a small number of alveolar epithelial cells with necrosis and hyperplasia (Fig. 4E, F).

### Immunohistochemical test results

Through immunohistochemical analysis of different tissues, the results showed that *M. ovipneumoniae* antigen was present in pathological sections of all visceral tissues, but the organs or tissues where *M. ovipneumoniae* was mainly distributed were lung, bronchus, and trachea. Among them, the average proportion of *M. ovipneumoniae* positive area in the lung, bronchus, and trachea was 5.30%, 3.87%, and 0.78% respectively. The percentage of positive *M. ovipneumoniae* in the lung was significantly different from the trachea (*P* = 0.0364).

**Fig. 5:**
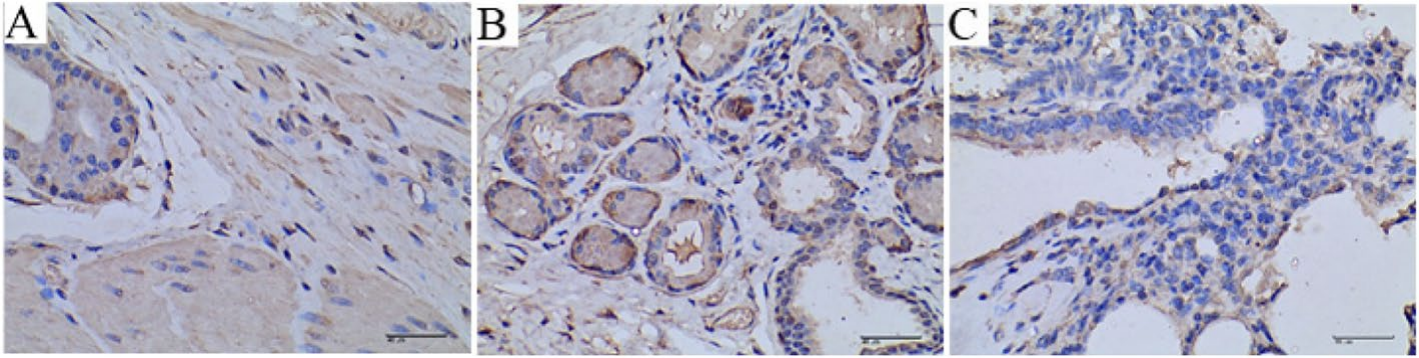
Immunohistochemical analysis of Lung, trachea, and bronchus (400 ×). **A** Histopathological analysis of trachea. **B **Histopathological analysis of bronchus. **C** Histopathological analysis of lungs. The *M. ovipneumoniae* antigen-positive region is shown in brown

Immunohistochemical sections of the lung showed alveolar cells, collapse, exfoliation and necrosis, and thickened alveolar septum. The area of lung tissue in the intercellular and visual field was about 196,603.10μm^2^, and the total area of *M. ovipneumoniae* positive staining was about 11,704.44μm^2^. The positive area accounted for about 5.95% (Fig. 5A).

Immunohistochemical analysis of bronchial sections showed that the cells in the upper bronchial lumen were exhaled and necrotic, and a large amount of *M. ovipneumoniae* was attached to the bronchial lumen, and a large amount of *M. ovipneumoniae* was colonized in the cytoplasm of epithelial cells. The bronchial tissue area in the visual field was about 196,607.90μm^2^, and the total area with *M. ovipneumoniae* positive staining was about 10,946.44μm^2^, accounting for about 5.57% of the positive area (Fig. 5B).

Epithelial cells were exfoliated in immunohistochemical sections of the trachea, and *M. ovipneumoniae* was found both on the surface of the epithelial cells and in the cytoplasm. The tracheal tissue area in the visual field was about 196608μm^2^, and the total *M. ovipneumoniae* positive staining area was about 2796.5μm^2^, accounting for about 1.42% of the positive area (Fig. 5C).

### Cytokine transcription level

**Fig. 6:**
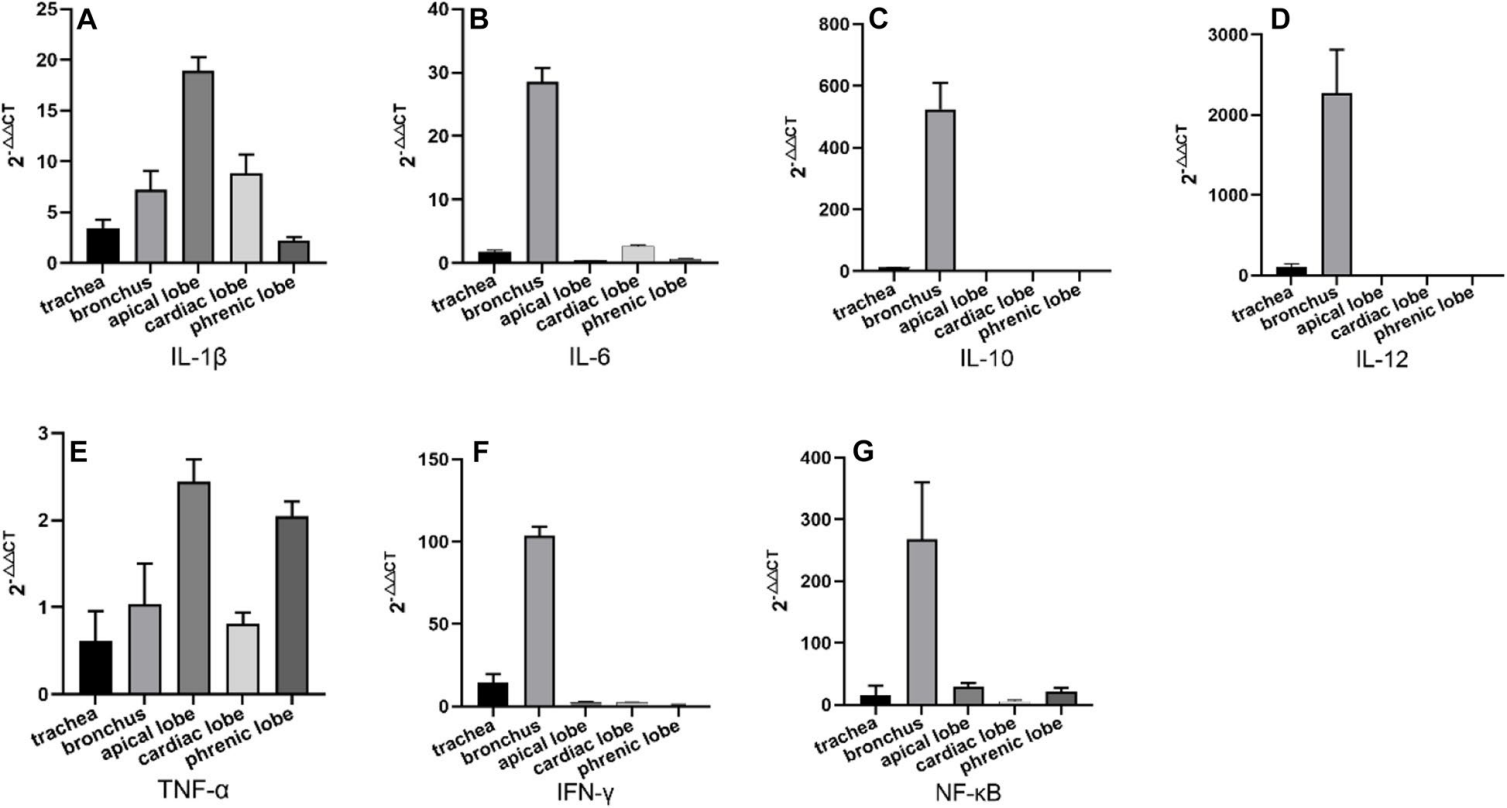
The mRNA expression of the same cytokine at different sites

The relative expression levels of cytokine mRNA were detected in the trachea, bronchus, lung, and its different sites by using qRT-PCR method. The results showed that the expression levels of IL-1β mRNA in the trachea, bronchus, and lung were increased, and the increasing degree was on the rise from the trachea to the apical lobe of the lung and on the decline from the apical lobe to phrenic lobe of the lung. IL-1β mRNA levels were positively correlated with the degree of lung lesions (Fig. 6A). The mRNA expression of IL-6 increased in trachea, bronchus and apical lobe, cardiac lobe of lung, and the most abundant expression in the bronchus (*P* < 0.01) compared with other tissues (Fig. 6B). The mRNA expression of IL-10 and IL-12 increased in trachea and bronchus, which was the most significantly up-regulated in bronchus (*P* < 0.01) compared with other tissues (Fig. 6C, D). The mRNA expression of TNF-α increased in the bronchi and all lobes of the lung, with the highest expression in the apical lobe of the lung (Fig. 6E). The expression of IFN-γ mRNA increased in the trachea and bronchus, which was the most significantly up-regulated in trachea and bronchi (*P* < 0.01) compared with other tissues (Fig. 6F). The mRNA expression of NF-κB increased in the trachea, bronchus and lung, with the highest expression in bronchus, which was the most significantly up-regulated in bronchus (*P* < 0.01) compared with other tissues (Fig. 6G).

**Fig. 7:**
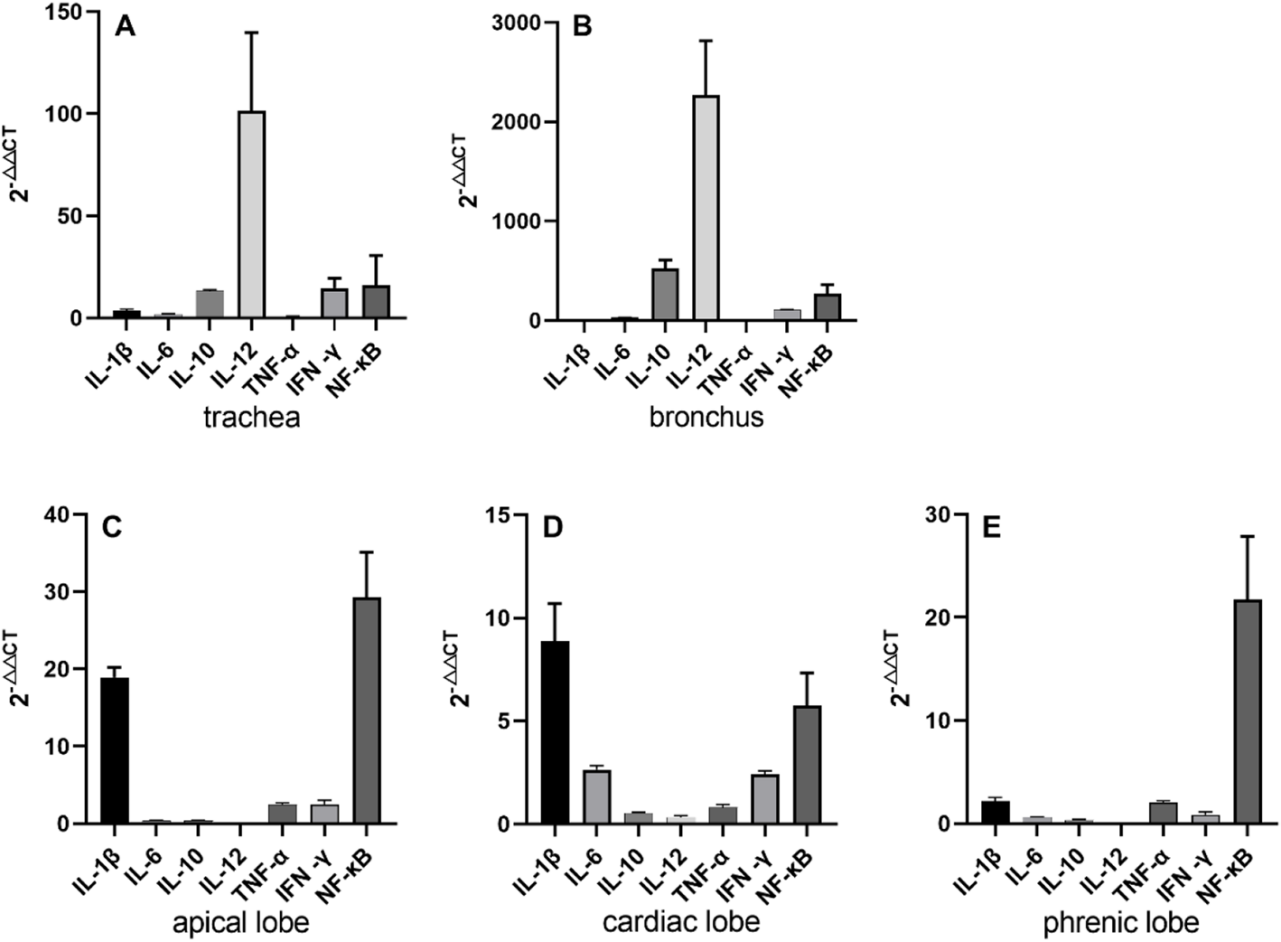
The mRNA expression of different cytokine mRNA at the same site

The expression of seven cytokines were detected in tracheal tissues following *M. ovipneumoniae* infection. IL-1β, IL-6, IL-10, IL-12, IFN-γ, and NF-κB were up-regulated and TNF-α was down-regulated (Fig. 7A). Cytokines up-regulated in bronchus included IL-1β, IL-2, IL-10, IL-12, TNF-α, IFN-γ and NF-κB (Fig. 7B). IL-1β, TNF-α, IFN-γ, and NF-κB were up-regulated expression in the apical lobe of the lung (Fig. 7C); IL-1β, IL-6, TNF-α, IFN-γ, and NF-κB were up-regulated expression in cardiac lobe of lung (Fig. 7D); IL-1β, TNF-α, and NF-κB were up-regulated in phrenic lobe of lung (Fig. 7E).

## Discussion

The diagnosis of *M. ovipneumoniae* infection mainly depends on the isolation and identification of pathogens, immunological detection, and molecular biological detection, which the isolation and culture of pathogens is the most basic method. In this study, no bacterial colony growth was observed on the solid medium of 5% defibrinated sheep blood, while typical mycoplasma "fried egg-like" colony morphology was observed on Thiaucourt’s solid medium, so it was initially identified as presumptive mycoplasma. The target product in PCR amplification using *M. ovipneumoniae*-specific primers was obtained, and the sequence analysis of the amplified product showed that it has the highest homology with *M. ovipneumoniae*.

Quantitative and qualitative analysis of *M. ovipneumoniae* antigen localization at the cellular level using immunohistochemistry demonstrated the presence of *M. ovipneumoniae* in the lung, trachea, bronchus, and other tissues. The percentage of positive *M. ovipneumoniae* in the lung, bronchus, and trachea was significantly different from the percentage of positive *M. ovipneumoniae* in the heart, liver, kidney, and spleen tissues, indicating that the amount of *M. ovipneumoniae* in lung, trachea, and bronchus was significantly more than that of other tissues, which is consistent with other related research reports [9, 14]. The percentage of positive *M. ovipneumoniae* in the lung was significantly different from the trachea (*P* = 0.0364). Immunohistochemical examination revealed a high load of *M. ovipneumoniae* antigens within lung lesions, which is consistent with Handeland et al. and Xue et al. [15, 16]. It further indicates that *M. ovipneumoniae* mainly infects tissues such as the lungs, trachea, and bronchi. Immunohistochemistry analysis showed that *M. ovipneumoniae* can colonize the surface of trachea and interstitial space, even inside cells, which was consistent with the conclusion reported by Kilic et al. [5] that *M. ovipneumoniae* can colonize epithelial cells and cytoplasm of trachea and lung. In this study, the lesions with strong positive signals in immunohistochemical pathological sections showed shrinkage and necrosis of alveolus cells, which was consistent with the test results of Zhang et al. [14].

IL-1β is an important regulatory substance involved in immune response and inflammatory reaction. Yang et al. [17, 18] found that large amounts of IL-1β can be secreted by myeloid monocyte cells, peripheral blood monocytes, and lung epithelial tumor cells during human mycoplasma pneumoniae infection. Pietsch et al. [19] study in the mouse model of mycoplasma pneumonia showed that IL-1β mRNA expression levels increased in the early and late stages of mycoplasma pneumoniae infection. As the first line of defense of the lung, the airway epithelium provides a physical barrier to prevent infection but also produces chemokines and cytokines such as TNF-α, IL-1β, IL-6, IL-8, and IL-12 that are important mediators in both lung defense and inflammation [20]. This study showed that IL-1β mRNA levels were up-regulated in the trachea, bronchi, and lung of *M. ovipneumoniae* infected sheep compared with healthy sheep. IL-1 can induce antigen-presenting cells to up-regulate the expression levels of MHCII molecules, various adhesion molecules, and IFN-γ receptors. Moreover, the pathogen molecular pattern can induce the secretion of IL-1 from macrophages Mφ, and IL-1 and TNF-α coordinate to mediate the acute immune response [21, 22]. The results of this study also showed that TNF-α mRNA was up-regulated in different tissues, indicating that IL-1 synergized with TNF-α to mediate the acute phase immune response after *M. ovipneumoniae* infection.

IL-6 is a cytokine that plays an important role in immune defense. Studies showed that IL-6 can be used as a sensitive indicator to identify early tissue injury and acute inflammatory reaction [23, 24]. In the study, the expression of IL-6 in the trachea, bronchus and apical lobe, cardiac lobe of the lung was up-regulated, indicating that IL-6 activated the defense system to participate in the inflammatory response in the process of *M. ovipneumoniae* infection.

The lack of IL-10 secretion can lead to various inflammation and even the persistence of inflammation and irreversible tissue damage [25–27]. In this study, the expression of IL-10 was the most significantly up-regulated in the bronchus and trachea significantly (*P* < 0.0001) compared with other tissues. However, IL-10 was not significantly up-regulated or down-regulated in the apical lobe, cardiac lobe, and phrenic lobe of the lung, which indirectly showed irreversible lesion.

IL-12 can promote the proliferation of lymphocytes and natural killer cells, stimulate the production of IFN-γ by T lymphocytes and natural killer cells, and enhance the cytotoxic function of natural killer cells [27–29]. In this study, we also found that IL-12 and IFN-γ were the most significantly up-regulated in the trachea and bronchi (*P* < 0.01) compared with other tissues, which showed that the production of IL-12 may promote the production of IFN-γ and then promote the cytotoxic function of natural killer cells.

NF-κB is the first responder to noxious cell stimulation, and NF-κB signaling is caused by extracellular stimuli [30]. In this study, NF-κB was upregulated in the trachea, bronchus, and lungs after *M. ovipneumoniae* infection, indicating that *M. ovipneumoniae* has completely invaded these organs, consistent with the immunohistochemical results. The cytokines of significantly up-regulated mRNA expression in the lung included IL-1β and NF-κB. There is a positive correlation between the changes in IL-1β and NF-κB. It may be that NF-κB, as an important transcription factor, participates in the Transcriptional regulation of IL-1β, which was consistent with Xue et al. [16, 31].

## Conclusion

*M. ovipneumoniae* is primarily found in the lungs of infected individuals. NF-κB, an essential transcription factor, is involved in the regulation of IL-1β transcription. IL-12 may enhance the cytotoxic function of natural killer cells during *M. ovipneumoniae* infection. Those findings demonstrate the distinct expression profiles of cytokines in various anatomical sites throughout disease progression, suggesting the potential role of bronchial tissue as a major site of immune response.

## Methods

### Samples collection

In November 2021, respiratory diseases occurred in 13 sheep farms in Ningxia. 5 diseased and dead sheep from 5 farms among 13 sheep farms were autopsied and the pathological organs were collected. One healthy sheep was euthanized by intravenous injection with 4% pentobarbital (1 mg/kg) and then organ and tissue samples were collected. A total of 105 samples were collected from 13 sheep farms (Table 1) and transported back to the laboratory at low temperatures for etiological diagnosis.

**Table 1:** Number of the samples collected from diseased sheep in different farms

Farms	No. of sheep	No. of infected sheep	Total samples	No. of sheep collected with nasal swab	No. of sheep collected with tissue
1	240	24	12	11	1
2	90	16	13	13	0
3	145	40	13	12	1
4	76	20	10	10	0
5	44	10	5	4	1
6	83	11	11	10	1
7	37	4	4	4	0
8	80	20	5	5	0
9	74	19	4	4	0
10	95	24	8	8	0
11	110	28	9	8	1
12	66	12	5	5	0
13	40	8	6	6	0
Total	1180	236	105	100	5

### Isolation and identification of *M. ovipneumoniae*

Thiaucourt's solid medium was used for the isolation of *M. ovipneumoniae* from the 13 representative samples from 13 farms. Blood agar including 5% defibrinated sheep blood was used to exclude other bacteria that can cause pneumonia. The inoculated media were aerobically incubated at 37 °C for 16–24 h. A single colony of each mycoplasma isolate was inoculated into Thiaucourt's liquid medium and incubated at 37 °C for 24 h before DNA extraction [2, 32].

DNA from 105 samples and 13 mycoplasma isolates was extracted using the Bacterial Genome DNA Extraction Kit (TIANGEN, Beijing, China) and was used as a PCR template. 16S rRNA of mycoplasma, specific genes of *M. ovipneumoniae*, and other mycoplasma were amplified to identify pathogen species by using specific primers in Table 2 [33, 34]. *M. ovipneumoniae* Y98 (ATCC 29419) was used as a quality control reference strain. PCR mixtures were prepared according to the instruction manual of the PCR Kit (TIANGEN, Beijing, China). Amplification conditions were performed according to the Kit instructions (TIANGEN, Beijing, China) (Table 2). PCR products were sequenced by Sangon Biotech (Shanghai) Co., Ltd. The phylogenetic tree was constructed and analyzed by the MEGA11.0 software (Neighbor-joining, NJ).

**Table 2:** Primer sequence information of different mycoplasmas

Primer name	Primer sequence	Amplified sequence length /bp	annealing temperature /℃
16S rRNA of Mycoplasma	M-16sF: TGCACCATCTGTCACTCTGTTAACCTC	1042	58
	M-16sR: AGAGTTTGATCCTGGGCTCAGGA		
*M. ovipneumoniae*	Mo-VPF: GTTGGTGGCAAAAGTCACTAG	418	53
	Mo-VPR: CTTGACATCACTGTTTCGCTG		
*M. mycoides* subsp. *capri*	MMC-F: ACTGAGCAATTCCTCTT	195	46
	MMC-R: TTAATAAGTCTCTATATGAAT		
*M. agalactiae*	Mag-F: CCTTTTAGATTGGGATAGCGGATG	360	60
	Mag-R: CCGTCAAGGTAGCGTCATTTCCTAC		
*M. arginini*	Ma-F: GCATGGAATCGCATGATTCCT	525	46
	Ma-R: GGTGTTCTTCCTTATATCTACGC		

### Histopathological observation

The pathological sections of the heart, liver, spleen, lung, kidney, trachea, and bronchus from 5 diseased sheep were made and stained with hematoxylin–eosin (H.E). 2 slices were observed for each tissue, at 100 × and 400 × , respectively [35]. The images of the slices were collected by digital slice scanner (3DHISTECH, Budapest, Hungary) and the specific pathological changes were photographed and characterized.

### Immunohistochemical detection

The pathological sections of the heart, liver, spleen, lung, kidney, trachea, and bronchus from 5 diseased sheep were repaired according to Rodriguez F. et al. [36], the first antibody (positive serum anti-*M. ovipneumoniae*), and the second antibody (rabbit anti-sheep IgG-HRP) were reacted, and the immunohistochemical specimens were made. The digital microphotography system was used to collect pictures of the histochemical sections. Each slice is 100 × ahead of the observation of all tissues, and then 3 microscopic images of each tissue are collected under 400 × . In the image, the nucleus was blue and the positive signal of *M. ovipneumoniae* was brown. The Halo data analysis system was used to calculate *M. ovipneumoniae* positive area and negative tissue area in each image.

### Cytokine detection

The main target organs, trachea, bronchus, apical lobe, cardiac lobe, and phrenic lobe of the lung, collected from affected sheep were used for pathological anatomy, histopathology analysis, and cytokine detection. The corresponding tissues from one healthy sheep were used as negative controls. Three samples of each visceral tissue were collected, and liquid nitrogen was frozen and transported back to the laboratory at ultra-low temperature. RNA was extracted with Trizol reagent (Takara, Osaka, Japan) and reverse transcribed into cDNA using Evo M-MLV Mix Kit (Accurate, Changsha, Hunan, China). Fluorescence quantitative PCR amplification was carried out with the primers in Table 3 [16, 31], with 3 repeats in each sample. The reaction system included 2 × SYBR Green PCR Master Mix 10μL, QN ROX Reference Dye 2μL, Forward Primer 1.4 μL(10 μmol/L), Reverse Primer 1.4 μL(10 μmol/L), cDNA < 100 ng and added ddH_2_O to 20μL. The reaction mixture was incubated at 95℃ for 2 min, 40 cycles of 95℃ for 5 s, 60℃ for 10 s. The mRNA transcription level of cytokines was detected and expressed by 2^−△△CT^. The internal reference is β-actin gene of sheep [16, 31].

**Table 3:** Primer sequence information of different cytokines

Primer name	Primer sequence	Amplified sequence length /bp
β-actin	F: AGAAGGCCAACATCCGGAACATGTCT	169
	R: CTTGATGGTGATGCAACGCTCCTGCT	
IL-1β	F: TCACCAGCTCTACAACAAA	105
	R: AGGTCATCATCACGGAAG	
IL-6	F: CTGCTCCTGGTGATGACTTCTGCTTT	132
	R: CGACGATGTGCTTAATGAGAGCTTCG	
IL-10	F: ACATGCTGCGGGACGTCCGAGCTGCCT	136
	R: CGACAAGGCTTGGCAACCCAGGTAAC	
IL-12	F: TTATCATCATGTTGCTGCTAGTTAAGG	156
	R: TTAAGACTGGAGGATGGCAAGTAGCCTT	
TNF-α	F: AGAAGTTGCTGGTGCCTCAGCCTCTT	125
	R: CAAGGCTGGCCAGAGACTCACCTCTT	
IFN-γ	F: TTAATGCAAGTAACCCAGATGTAGCT	185
	R: ATGTCTTGCTTGATGATATCCATGC	
NF-κB	F: CGAGGATGATGAGAATGG	133
	R: CAGGAACACGGTTACAGG	

### Statistical analysis

One-way ANOVA was used for comparisons of the mean between different groups by GraphPad Prism 7 (GraphPad Software, San Diego, California, USA). Pearson's correlation coefficient was applied to analyze the correlation of different cytokines.

### Supplementary Information

**Additional file 1: Supplementary Data 1.** Immunohistochemical analysis of lung, bronchus and trachea.

**Additional file 2: Figure S1.** PCR results of isolates.

**Additional file 3: Figure S2.** Histopathological analysis of trachea (100x).

**Additional file 4: Figure S3. **Histopathological analysis of trachea (400x).

**Additional file 5: Figure S4. **Histopathological analysis of bronchus (100×).

**Additional file 6: Figure S5. **Histopathological analysis of bronchus (400×).

**Additional file 7: Figure S6. **Histopathological analysis of lung (100×).

**Additional file 8: Figure S7. **Histopathological analysis of lung (400×).

## Data Availability

All data generated or analyzed during this study are included in this article. The datasets generated and analyzed in this study are available in the National Center for Biotechnology Information repository (OQ652949-OQ652961).

## Abbreviations

NK: Natural killer
IL: Interleukin
TNF: Tumor necrosis factor
IFN: Interferon
NF: Nuclear factor
MHC: Major histocompatibility complex
HRP: Horse radish peroxidase
H.E.: Hematoxylin–eosin
