# Supplementary figures and images for: Analysis of histopathology and changes of major cytokines in the lesions caused by Mycoplasma ovipneumoniae infection
Source: BMC Vet Res. 2023 Dec 15;19:273. doi: 10.1186/s12917-023-03829-4 (PMC10722778; doi:10.1186/s12917-023-03829-4)

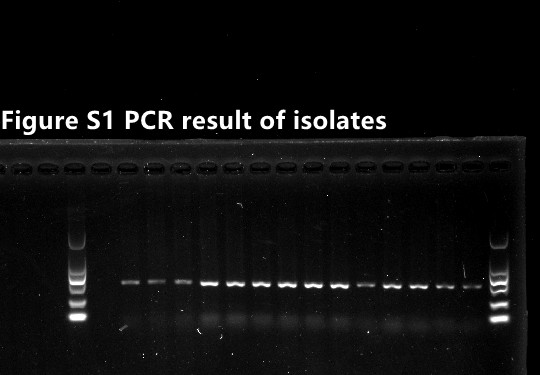

Supplement: Supplementary file 2 — Additional file 2: Figure S1. PCR results of isolates. [file 12917_2023_3829_MOESM2_ESM.jpg]

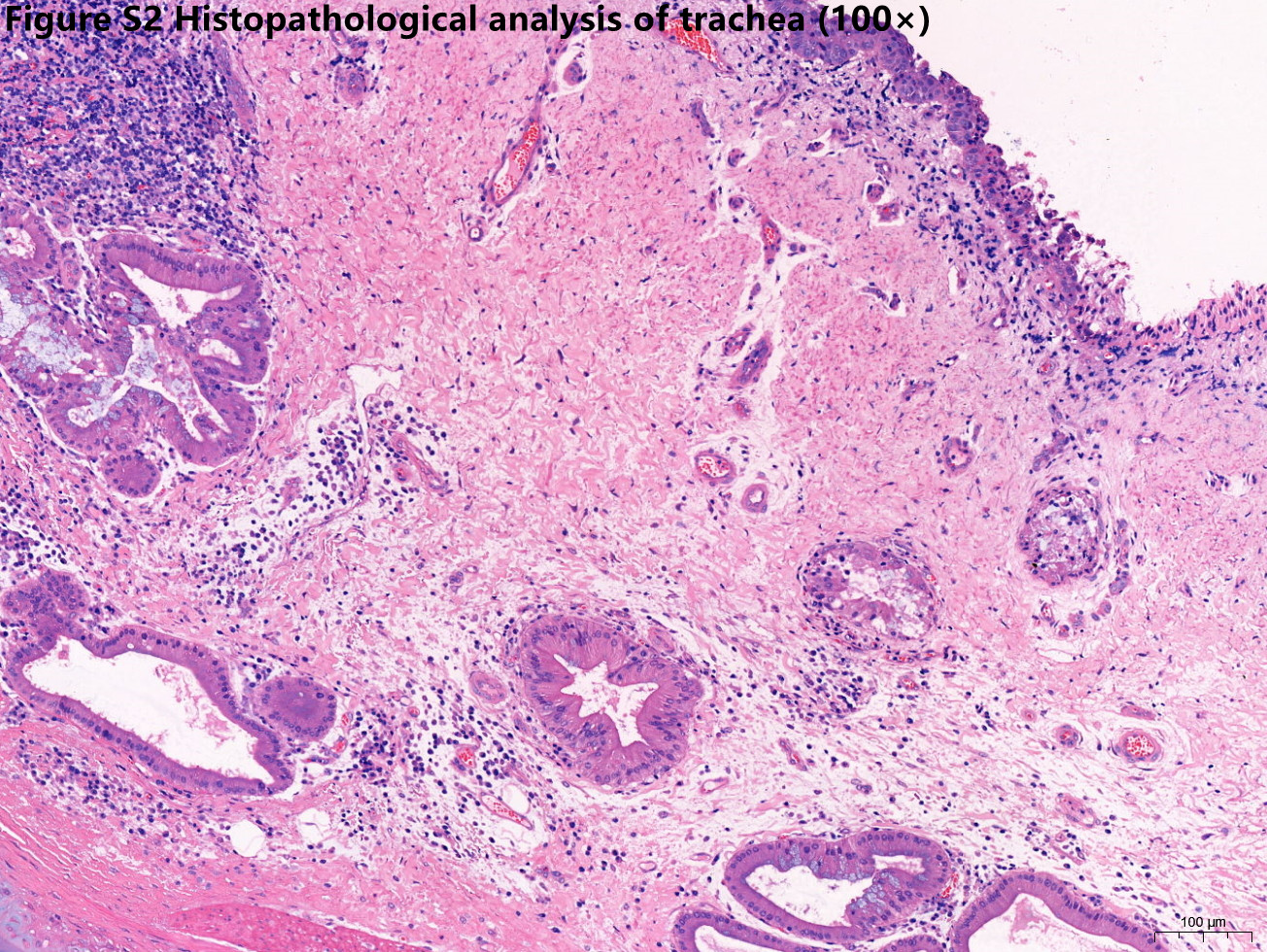

Supplement: Supplementary file 3 — Additional file 3: Figure S2. Histopathological analysis of trachea (100x). [file 12917_2023_3829_MOESM3_ESM.jpg]

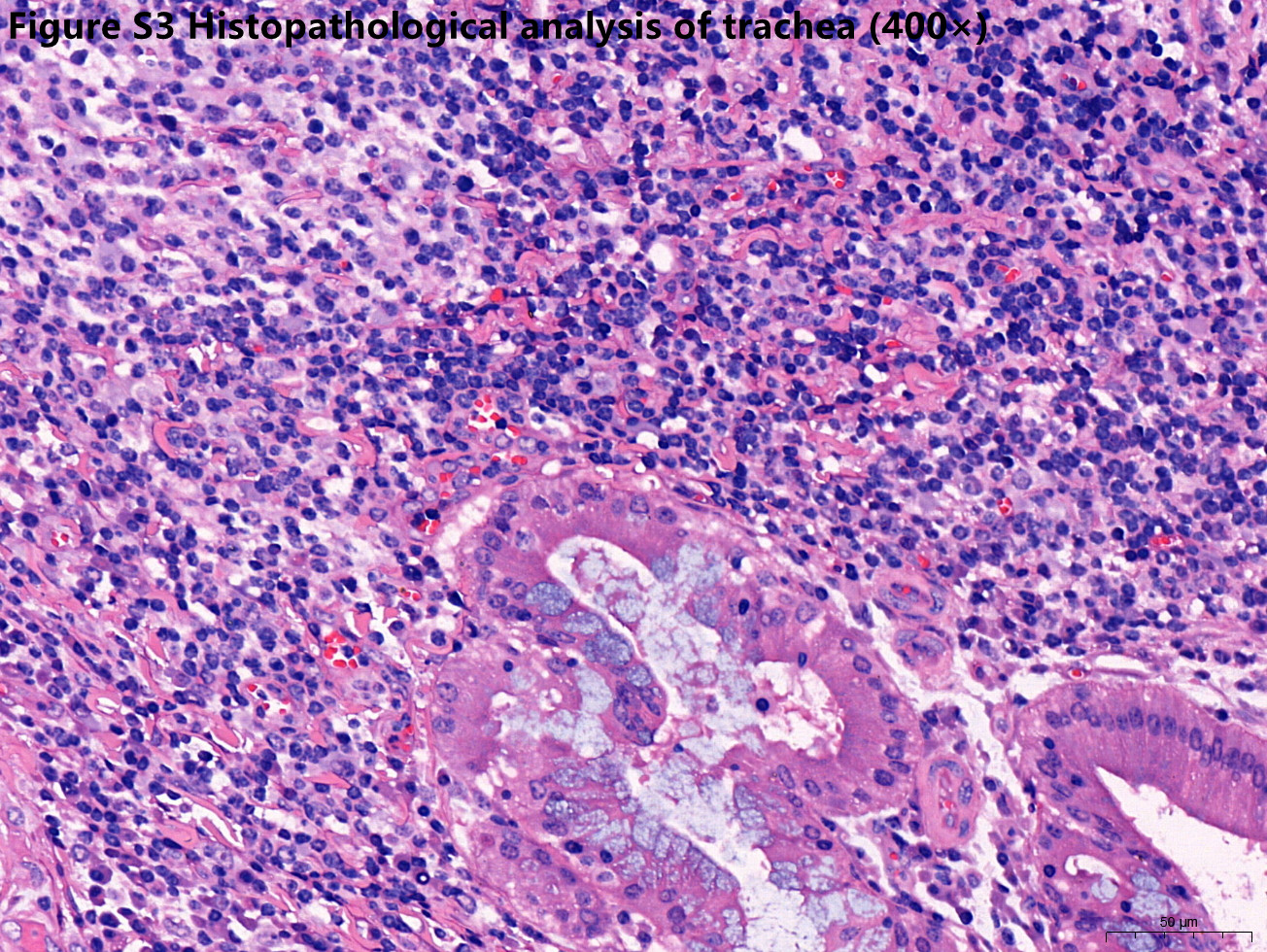

Supplement: Supplementary file 4 — Additional file 4: Figure S3. Histopathological analysis of trachea (400x). [file 12917_2023_3829_MOESM4_ESM.jpg]

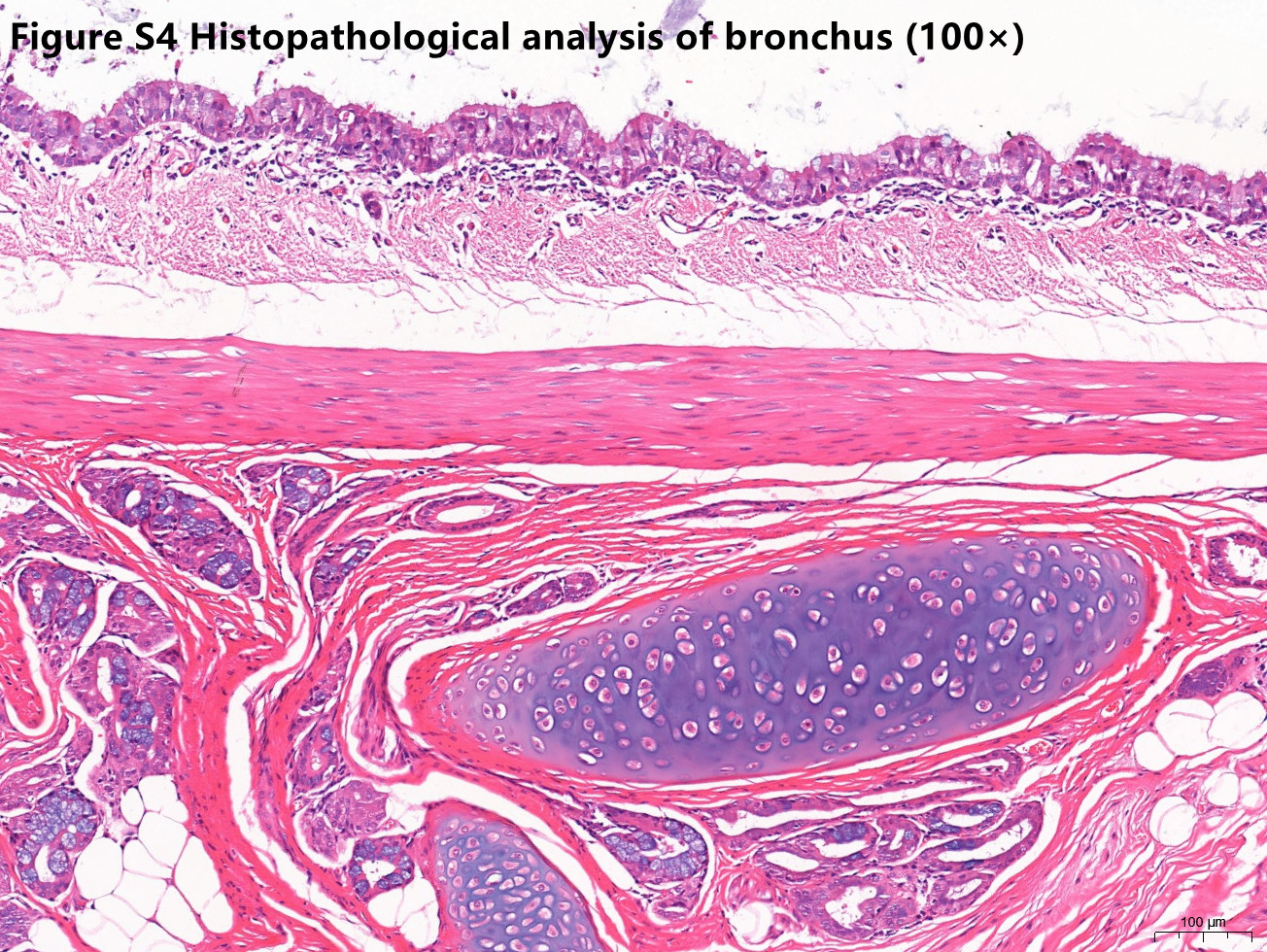

Supplement: Supplementary file 5 — Additional file 5: Figure S4. Histopathological analysis of bronchus (100×). [file 12917_2023_3829_MOESM5_ESM.jpg]

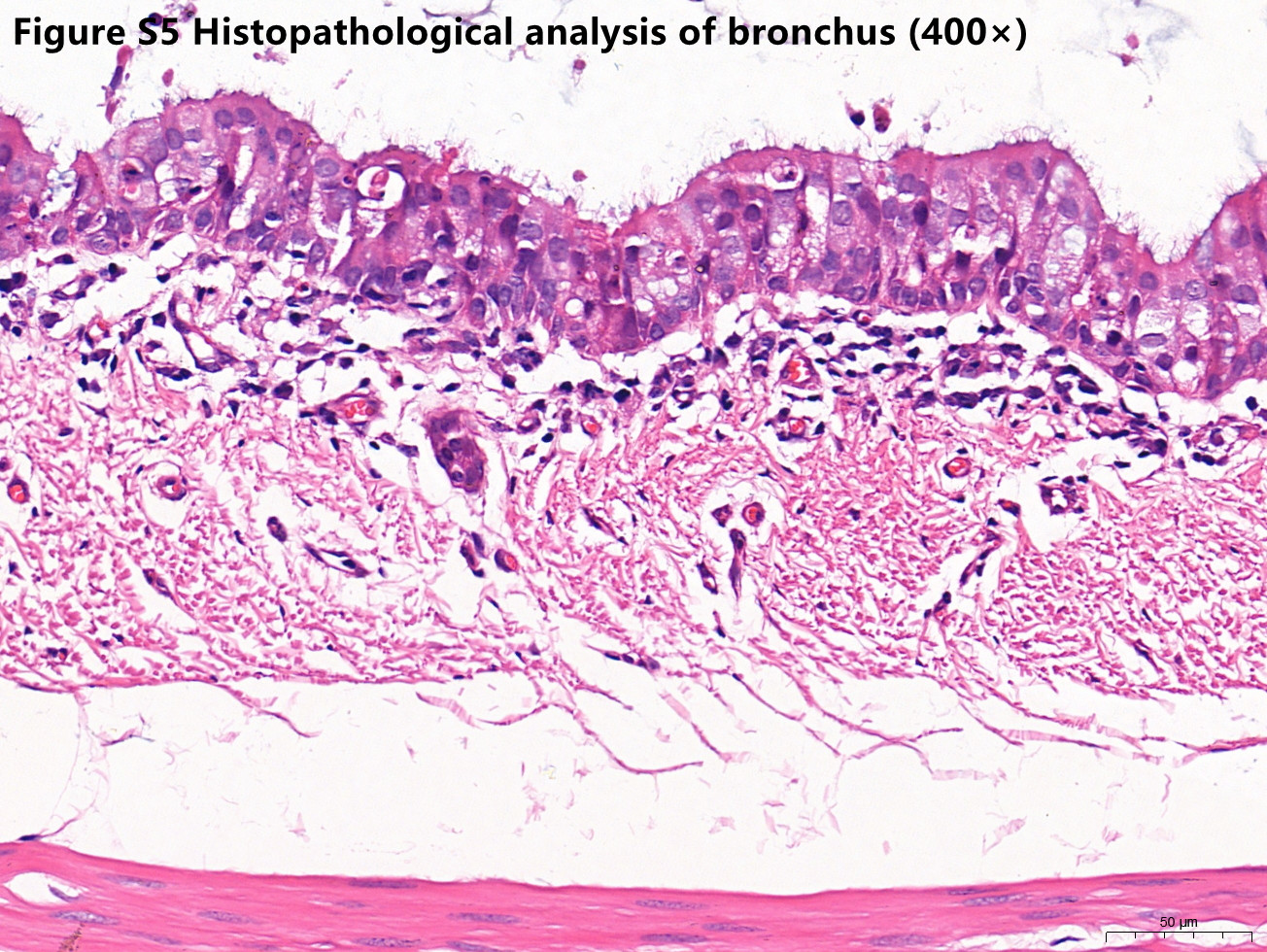

Supplement: Supplementary file 6 — Additional file 6: Figure S5. Histopathological analysis of bronchus (400×). [file 12917_2023_3829_MOESM6_ESM.jpg]

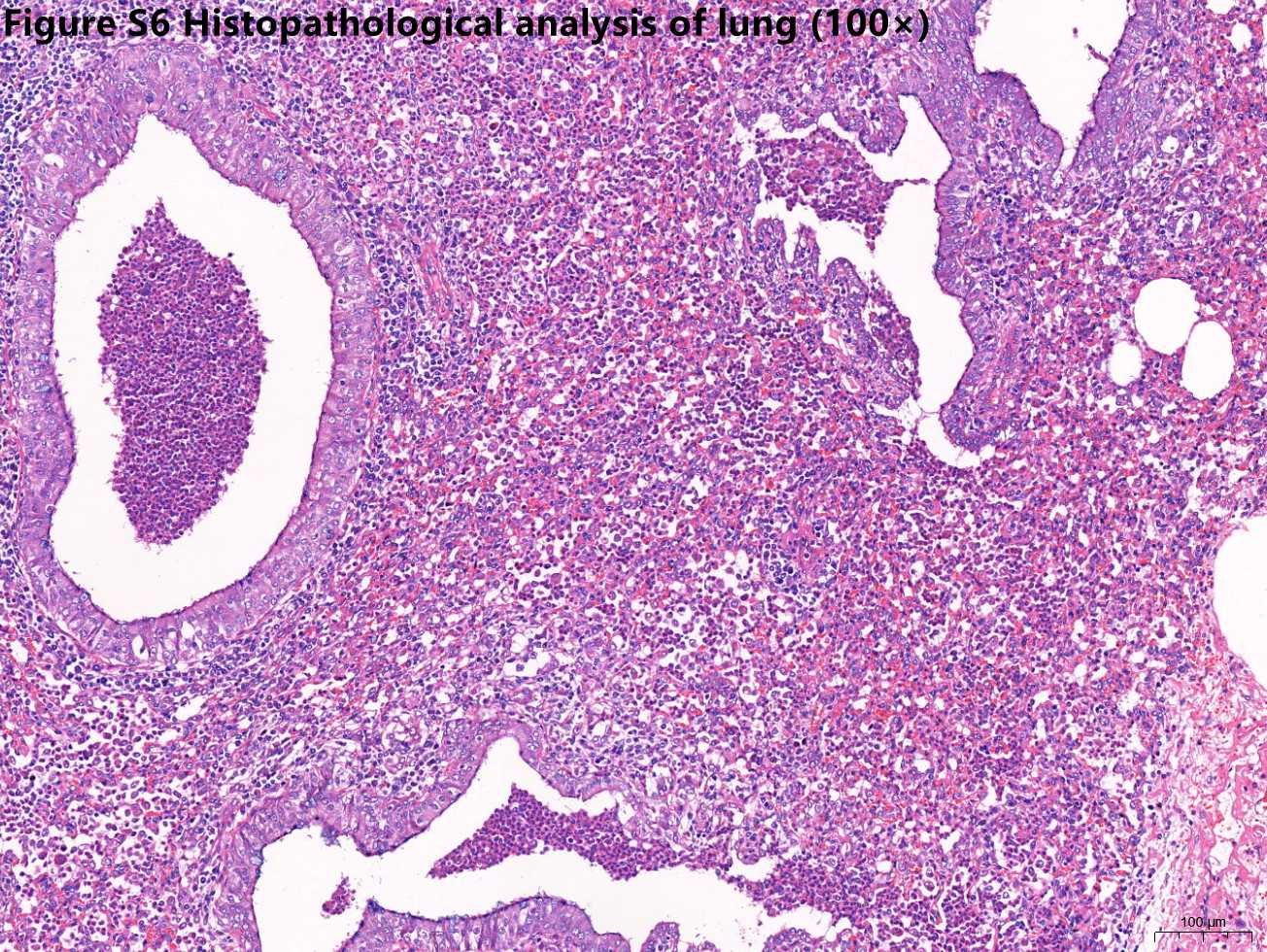

Supplement: Supplementary file 7 — Additional file 7: Figure S6. Histopathological analysis of lung (100×). [file 12917_2023_3829_MOESM7_ESM.jpg]

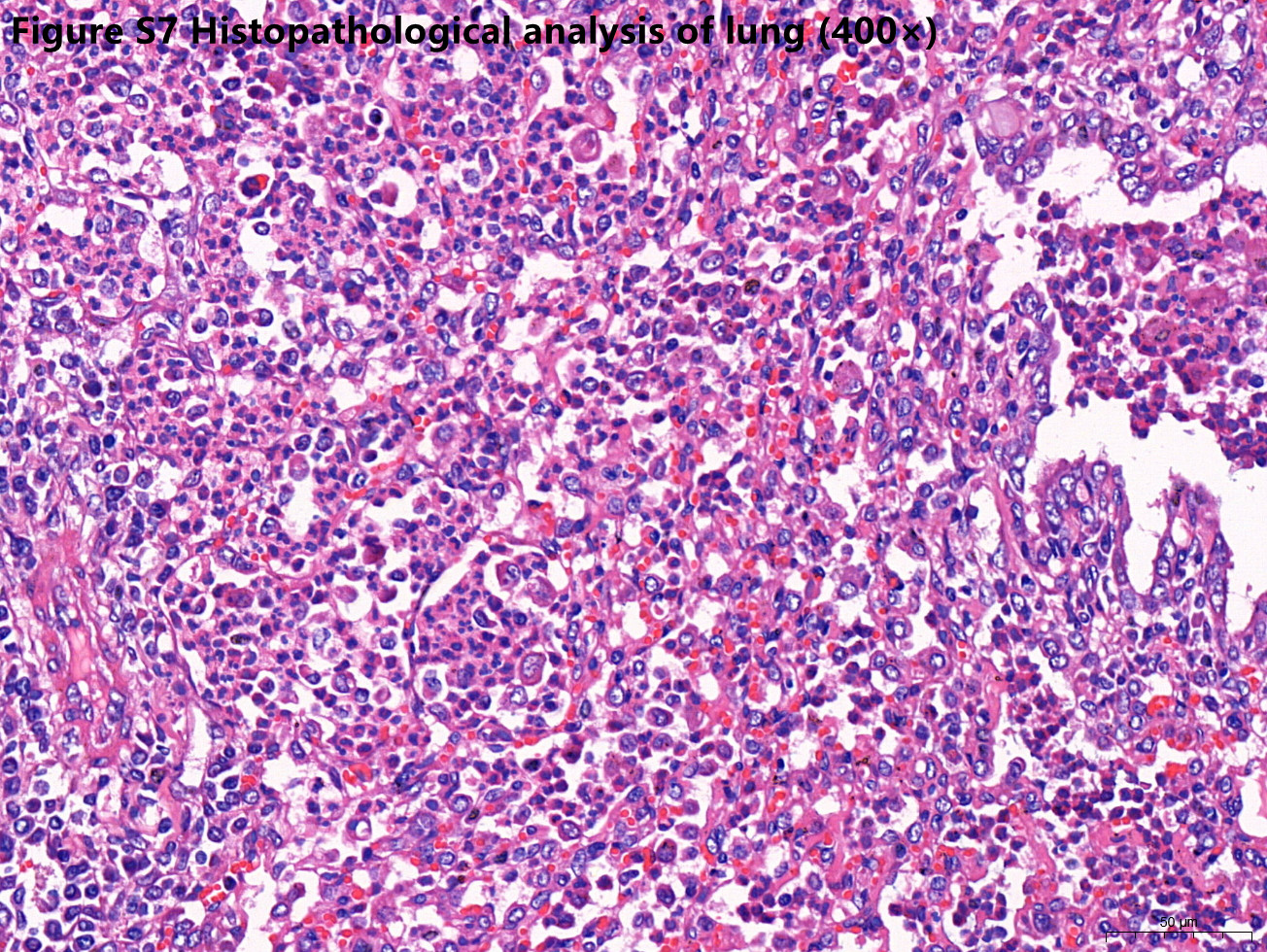

Supplement: Supplementary file 8 — Additional file 8: Figure S7. Histopathological analysis of lung (400×). [file 12917_2023_3829_MOESM8_ESM.jpg]
